# Large Bowel Obstruction, a Delayed Complication of Severe Gallstone Pancreatitis

**DOI:** 10.1155/2016/1034929

**Published:** 2016-10-26

**Authors:** Neeraj Lal, John Whiting, Rahul Hejmadi, Sudarsanam Raman

**Affiliations:** ^1^Queen Elizabeth Hospital, Birmingham, UK; ^2^Apollo Hospitals, Chennai, India

## Abstract

Colonic complications are rare after acute pancreatitis but are associated with a high mortality. Possible complications include mechanical obstruction, ischaemic necrosis, haemorrhage, and fistula. We report a case of large bowel obstruction in a 31-year-old postpartum female, secondary to severe gallstone pancreatitis. The patient required emergency laparotomy and segmental bowel resection, as well as cholecystectomy. Presentation of obstruction occurs during the acute episode or can be delayed for several weeks. The most common site is the splenic flexure owing to its proximity to the pancreas. Initial management may be conservative, stenting, or surgical. CT is an acceptable baseline investigation in all cases of new onset bowel obstruction. Although bowel obstruction is a rare complication of pancreatitis, clinicians should be aware of it due to its high mortality. Obstruction can occur after a significant delay following the resolution of pancreatitis. Those patients with evidence of colonic involvement on pancreatic imaging warrant further large bowel evaluation. Bowel resection may be required electively or acutely. Colonic stenting has an increasing role in the management of large bowel obstruction but is a modality of treatment that needs further evaluation in this setting.

## 1. Introduction

Colonic complications of pancreatitis including paralytic ileus, colonic necrosis, and pancreatic-colonic fistulae have been well described and occur with varying frequency [1]. However, mechanical obstruction of the colon due to pancreatitis is rare [2], and, to date, less than 30 cases have been reported in the literature. In this report, we describe a case of large bowel obstruction as a consequence of severe gallstone pancreatitis in a postpartum patient.

## 2. Presentation of Case

**Figure 1:**
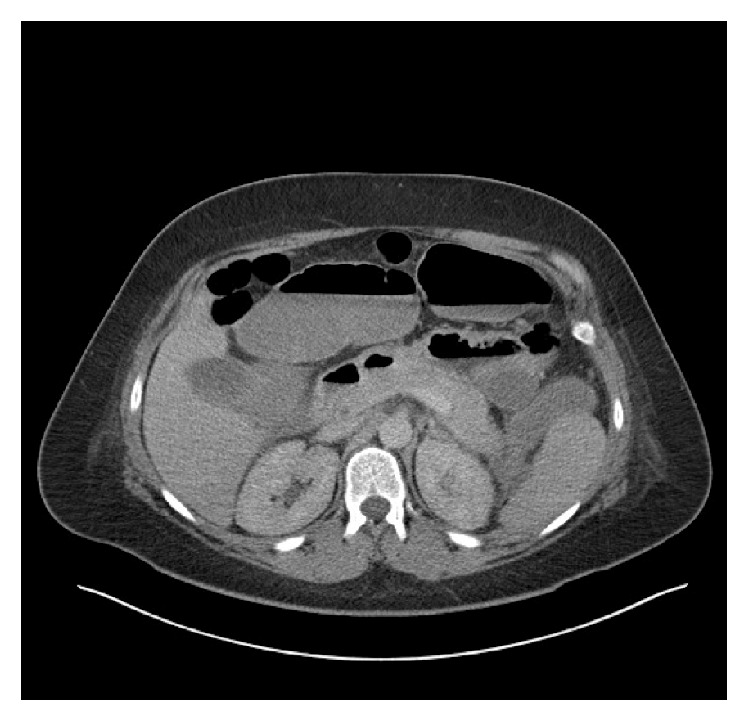
CT scan showing acute pancreatitis and bowel dilatation.

**Figure 2:**
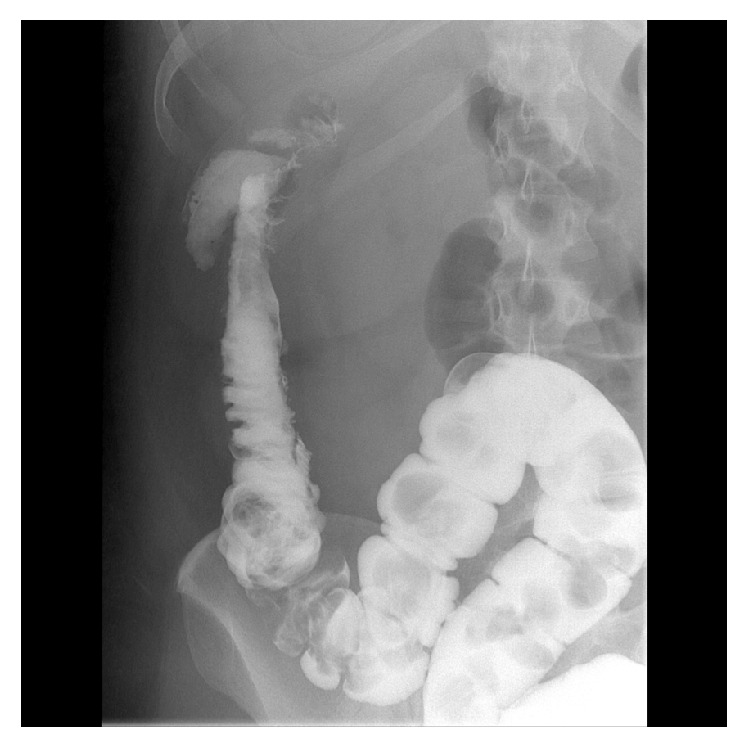
Gastrografin enema demonstrating stricture at splenic flexure region.

A 31-year-old female presented as an emergency to our department with a short history of painful abdominal distension and constipation. She had a recent admission with severe postpartum gallstone pancreatitis. A computed tomography (CT) scan during that admission showed pancreatitis with large bowel dilatation up to the splenic flexure and peripancreatic fluid collections (Figure 1). A subsequent gastrografin enema revealed a possible inflammatory stricture with localised perforation at the splenic flexure (Figure 2). She responded to conservative management and her bowels began to open. She was awaiting an interval cholecystectomy.

**Figure 3:**
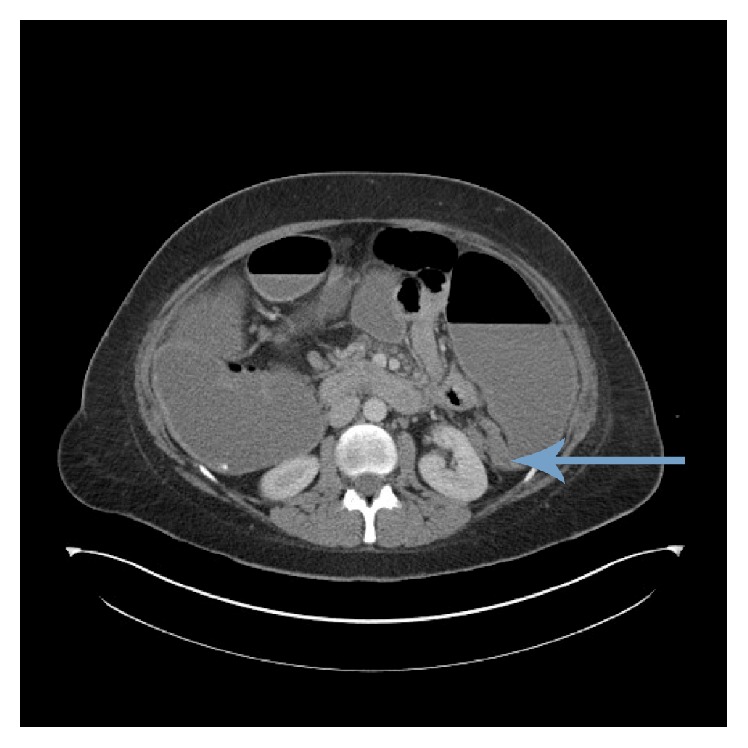
CT scan demonstrating large bowel obstruction with arrows showing cut-off at splenic flexure.

On readmission, she was noted to be anxious and clinically appeared dehydrated. Abdominal examination revealed distension with epigastric tenderness and absent bowel sounds. Blood tests were unremarkable. An urgent CT scan confirmed large bowel obstruction with a cut-off at the splenic flexure (Figure 3).

At laparotomy, it was noted that the large intestinal obstruction was due to a densely adherent inflammatory peripancreatic mass with surrounding fat necrosis. A segmental colonic resection was performed with primary stapled anastomosis. A retrograde cholecystectomy was also performed at the same time.

Her postoperative recovery was complicated by an anastomotic leak necessitating a relaparotomy and exteriorisation of the proximal colon. She made a slow but satisfactory recovery.

**Figure 4:**
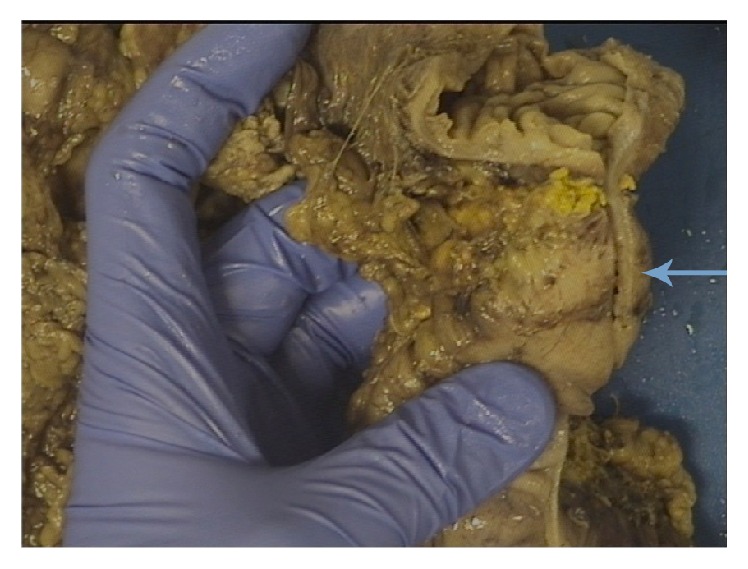
Segment of resected colon with the arrow showing the site of obstruction.

**Figure 5:**
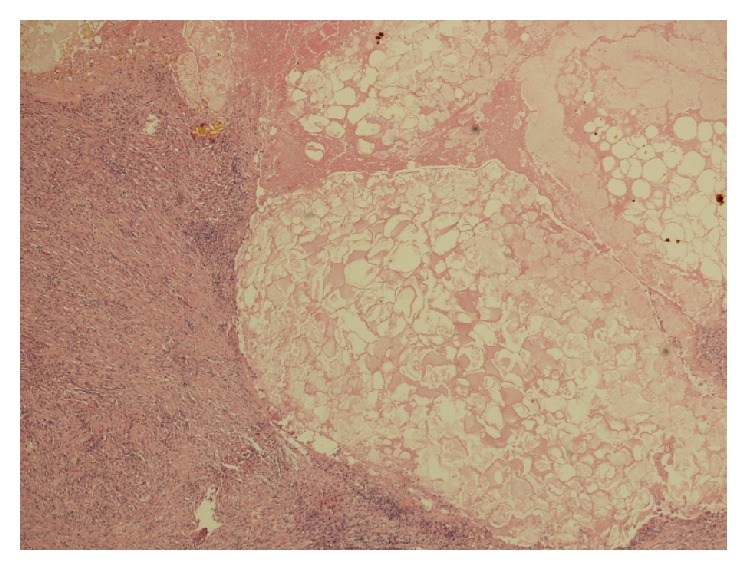
Histology showing pericolonic scarring and inflammatory changes around foci of pancreatic fat necrosis.

Gross pathological examination (Figure 4) showed the resected segment of colon with a tight stricture measuring 2 cm in length. The stricture comprised a rim of scarred fatty tissue around the colonic wall with intact mucosa. Microscopic examination revealed dense pericolonic lymphocytic and histiocytic inflammatory response with areas of fibrosis centred on distinct areas of pancreatic fat necrosis, compressing the colonic wall (Figure 5). The mucosa, submucosa, and the muscularis propria of the colon were normal. The gall bladder specimen showed cholelithiasis and cholesterolosis.

## 3. Discussion

Colonic complications from pancreatitis are rare but are associated with substantial mortality and morbidity [3–5]. These complications include bowel obstruction, ileus, bowel necrosis, fistulae, and perforation [6]. The exact frequency of these complications is unclear. A retrospective review of 296 patients revealed that 6.1% developed colonic complications. Only one case had incomplete colonic stenosis [7]. Presentation with complete intestinal obstruction is uncommon.

Recognition of large bowel involvement is difficult as symptoms may be nonspecific or masked by systemic features of pancreatitis [4]. The development of obstruction has been reported during the acute episode of pancreatitis and during the weeks after recovery [5, 6, 8, 9].

Many pathological hypotheses have been suggested for development of colonic obstruction following pancreatitis. External compression by the inflamed mesocolic mass can lead to necrosis of fatty tissue [10]. Fat necrosis is the result of the enzymatic action of lipase, released in pancreatitis [11]. The resultant fatty acids then complex with calcium to form deposits [12]. Additionally, the peritoneal reflections from the anterior surface of the pancreas provide a route for the spread of both pancreatic enzymes and inflammatory mediators within the transverse mesocolon and small bowel mesentery [6]. This may lead to fat necrosis and fibrosis, narrowing the bowel lumen. This provides an explanation for why the stenosis frequently occurs in the splenic flexure region, which is in close proximity to the pancreas. Additionally, the splenic flexure is a watershed region between the areas of supply of the middle and left colic arteries and is particularly sensitive to periods of hypotension during acute pancreatitis, leading to an ischaemic response [6]. It is likely that these pathological mechanisms account for the majority of colonic complications of pancreatitis.

Postoperative histology is useful to exclude primary large bowel pathology such as inflammatory bowel disease and neoplasia. In this case, histology confirmed the presence of scarring and pericolonic inflammatory changes secondary to pancreatic fat necrosis. Of note, though the initial gastrografin enema suggested the presence of a perforation, histology revealed an intact bowel wall. Gastrografin enema is very sensitive for the identification of radiological leak. However, as the patient responded to conservative management, it is unlikely that a significant clinical leak was present. Furthermore, as there was a lag period between the patient's gastrografin study and subsequent readmission and laparotomy, in the interim period a small perforation could have been sealed by fibrosis related to the inflammatory response. Consistent with this, the histology from the colonic resection revealed dense inflammatory infiltrates.

Initial management of large bowel obstruction following pancreatitis should follow that of all cases of intestinal obstruction, with optimal fluid resuscitation and frequent review. CT has replaced contrast enema in the investigation of acute large bowel obstruction and has become a standard and acceptable baseline modality to both diagnose and characterise patients with symptoms suggestive of obstruction [13]. With increasing familiarity with the technique, CT imaging's sensitivity for large bowel obstruction has surpassed that of contrast enema [14, 15].

Nonoperative approach may include the placement of a colonic stent. The majority of the evidence for the use of colonic stenting is in the malignant setting [16]. Its use in benign disease remains a controversial area [17]. Technical advances have allowed the use of stents in the splenic flexure region [18], but there is no reported use of it in pancreatitis related disease. Experience with other benign disease suggests that insertion of self-expanding stents is a safe procedure but surgery is required in a large number of cases due to primary or secondary failure [17]. Use of stents in cases of pancreatitis could be used as a temporising measure until the inflammation and obstruction improve.

In those patients in whom conservative measures fail, surgery with resection of the stenosed section of bowel will be required. In this case, segmental colectomy followed by primary anastomosis was performed initially. Intraoperatively, it was felt that primary anastomosis was appropriate given the patient's good physiological status and age, as well as healthy and viable appearing bowel edges together with good vascularity and mobility. The available evidence suggests that segmental resection and primary anastomosis are an acceptable option in large bowel obstruction [19]. This is highlighted in The Association of Coloproctology of Great Britain and Northern Ireland (ACPGBI) consensus statement for malignant large bowel obstruction [20]. Similar principles would also apply in the benign setting. There is a lack of data for primary anastomosis compared to colostomy in the splenic flexure region. However, the overall documented leak rate for segmental colectomy with or without on-table lavage following large bowel obstruction is roughly 4% [21–23].

In patients in whom pancreatitis was caused by gallstones, cholecystectomy is suggested to reduce the risk of further pancreatitis [24]. Cholecystectomy concurrent with the bowel resection, as was performed in this case, is appropriate to reduce the need for further surgery.

## 4. Conclusion

Large bowel obstruction is a rare complication of acute pancreatitis but one that clinicians should be aware of due to its high mortality. Obstruction can occur after a significant delay following the resolution of pancreatitis. Those patients with evidence of colonic involvement on pancreatic imaging warrant further large bowel evaluation. Bowel resection may be required electively or acutely. Large multicentred data series are needed to determine optimum management. Colonic stenting has an increasing role in the management of large bowel obstruction but is a modality of treatment that needs further evaluation in this benign setting.
